# Activation of AMP-activated protein kinase by metformin protects human coronary artery endothelial cells against diabetic lipoapoptosis

**DOI:** 10.1186/s12933-014-0152-5

**Published:** 2014-11-13

**Authors:** Linnéa Eriksson, Thomas Nyström

**Affiliations:** Department of Clinical Science and Education, Section of Endocrinology and Diabetology, Karolinska Institutet, Södersjukhuset AB, Stockholm, Sweden; Department of Molecular Medicine and Surgery, Karolinska Institutet, Karolinska University Hospital, Center for Molecular Medicine, Stockholm, Sweden

**Keywords:** Metformin, Endothelial cell, Free fatty acids, Apoptosis

## Abstract

**Background:**

The prevalence of type 2 diabetes (T2D) among adults worldwide is rapidly increasing, and in patients with diabetes the major cause of death is macrovascular disease. Endothelial cells play an important role in maintaining vascular homeostasis. Free fatty acids, which are elevated in T2D, have previously been shown to induce endothelial dysfunction and apoptosis of endothelial cells, which is considered as an important and early factor in the onset of atherosclerosis and cardiovascular disease. Metformin, which is used as first line treatment of T2D patients, is believed to exert its pharmacological effects through activation of AMP-activated protein kinase, which has emerged as a new potential target in reversing endothelial dysfunction.

**Methods:**

Here we studied the protective effect of metformin against free fatty acid-induced apoptosis of human coronary artery endothelial cells (HCAECs) by assessing DNA fragmentation and cleaved caspase 3 levels. We also attempted to elucidate the underlying mechanisms by investigating the involvement of AMP-activated protein kinase, p38 MAPK and eNOS. Generation of reactive oxygen species by free fatty acid exposure was also examined.

**Results:**

Our results suggest that metformin protects HCAECs from lipoapoptosis, an effect that involves eNOS and p38 MAPK, downstream of AMPK signaling, but not as previously suggested through suppression of reactive oxygen species.

**Conclusion:**

The protective effect of metformin against free fatty acid induced apoptosis is potentially clinically relevant as metformin is first line treatment for patients with T2D, a patient group which is rapidly increasing and carries a high burden of cardiovascular disease.

## Background

It is estimated that 382 million adults worldwide are suffering from diabetes today, a number which is projected to rise with more than 50% over the next 25 years [1]. In patients with diabetes, the major cause of death is macrovascular disease, and in individuals with type 2-diabetes (T2D), the main etiology for up to 75% of the mortality is atherosclerotic cardiovascular disease [2]. Controlling hyperglycemia *per se* has met with limited success in curbing macrovascular morbidity in T2D [3], highlighting the need to find agents effective in this respect.

The endothelium, the innermost lining of blood vessels, is situated as a barrier between the circulating blood and the tissue. Endothelial nitric oxide synthase (eNOS) controls the vascular tone in response to several stimuli by producing nitric oxide (NO). NO plays an essential role in the regulation of endothelial function [4]. Loss of physiological features of the endothelium, such as its preference to support vasodilatation, fibrinolysis and antiaggregation, is referred to as endothelial dysfunction [5]. It is oftentimes associated with loss of NO availability and precedes the development of atherosclerosis. This is observed in diabetic and obese individuals, and the extent of endothelium-dependent vasodilatation correlates with an individual’s insulin sensitivity [4]. Endothelial dysfunction has thus emerged as an important early target for preventing atherosclerosis and cardiovascular disease [6].

Free fatty acids (FFAs), formed during lipolysis from triglycerides, are known to impair the endothelial-dependent vasodilatation [7]; and elevated levels of FFAs are commonly seen in patients with T2D. FFAs decrease the bioavailability of NO and induce apoptosis of endothelial cells, two processes that are assumed to be involved in, and contribute to, endothelial dysfunction and atherosclerosis [7,8].

In the UK Prospective Diabetes Study (UKPDS), monotherapy with metformin decreased the incidence of macrovascular morbidity in overweight T2D patients. This outcome appeared to be independent of its glucose lowering property [9]. Metformin is believed to exert its pharmacological effects through activation of AMP-activated protein kinase (AMPK) [5]. AMPK has emerged as a new potential target not only in controlling glycemia, but also in reversing endothelial dysfunction. Activation of AMPK by metformin has been demonstrated to lead to phosphorylation of eNOS, thus stimulating the release of NO, which is paramount for vascular function [5,10]. In addition to this, activation of AMPK by 5-aminoimidazole-4-carboxamide-1-β-D-ribofuranoside (AICAR) has been shown to protect from palmitate-induced apoptosis through suppression of reactive oxygen species (ROS) production in bovine aortic endothelial cells [11]. Our aim was therefore to investigate the putative protective effect of metformin against diabetic lipoapoptosis in human coronary artery endothelial cells (HCAECs) and attempt to elucidate the mechanisms involved in imparting such an effect.

## Methods

### Cell culture

Normal primary HCAECs, isolated from normal human coronary arteries (passage 5–13) and obtained from Clonetics (Lonza, Walkersville, MD), were grown in EGM-2 MV medium supplemented with 5 mM glucose, hydrocortisone, human epidermal growth factor, 5% FBS, vascular endothelial growth factor, human fibroblast growth factor-B, R3-IGF-1, ascorbic acid and gentamicin/amphotericin-B at 37°C in a humidified atmosphere (5% CO_2_, 95% air) as recommended by the supplier. Cell culture experiments were approved by the local research ethics committee (232/03). Confluent cultures were detached by trypsin-2-[2-(bis(carboxymethyl) amino) ethyl-(carboxymethyl) amino]acetic acid and seeded onto tissue culture dishes and allowed to attach overnight before further investigations were performed.

To examine the effect of metformin on apoptosis, HCAECs were incubated in EGM medium containing 5% FBS and 2 mM L-glutamine. Metformin (100 μM-2 mM) [10,12,13], AICAR (200 μM), Compound C (10 μM), N_ω_-nitro-L-arginine methyl ester hydrochloride (L-NAME, 1 mM) (Sigma-Aldrich, St Louis, MO), p38 MAPK inhibitor SB203580 (10 μM) (Cell Signaling Technology, Danvers, MA), JNK inhibitor SP600125 (5 μM) (Calbiochem, La Jolla, CA) or vehicle were added 1 h prior to palmitate (125 μM) and were continuously present during the whole incubation.

### DNA fragmentation

HCAECs were seeded onto 6 well plates and incubated for 24 h in EGM medium containing 5% FBS and 2 mM L-glutamine, in the presence or absence of palmitate or vehicle, with or without metformin, AICAR, Compound C, L-NAME, SB203580, or SP600125. DNA fragmentation, a marker of apoptosis, was assayed by the cell death detection kit ELISA plus (Roche Diagnostics Scandinavia AB, Stockholm, Sweden), according to the manufacturer’s instructions. The ELISA measures cytoplasmic DNA-histone complexes that increase during apoptosis-associated DNA fragmentation.

### siRNA silencing of AMPK and quantitative PCR

HCAECs were seeded into 6 well plates and incubated for 24 h at 37°C in complete medium. Control siRNA and AMPKα1/2 siRNA (final concentration 10 nM, Santa Cruz Biotechnology, Heidelberg, Germany) was mixed and incubated with SilenceMag according to the standard protocol by the supplier (Oz Biosciences, Marseille, France). The siRNA/SilenceMag mix was then added drop-wise onto the cells and incubated on a magnetic plate for 15 min, the magnet was the removed from the culture plate and the cells were left in serum free condition for 3 h before supplementation with 5% FBS. Incubation for the evaluation of palmitate induced apoptosis started 48 h post transfection (as described above) and continued for 24 h.

48 h post transfection was protein and RNA isolated from separate wells for verification of gene silencing with western blotting (method described below) and quantitative PCR (qPCR). RNA was prepared using Qiazol Lysis Reagent (Qiagen, Hilden, Germany) and purified by RNeasy Mini kit (Qiagen, Hilden, Germany), including DNase digestion. The concentration and quality was measured using Nanodrop ND-1000 (Thermo Scientific, Waltham, MA). For qPCR, total RNA was reverse-transcribed using High Capacity RNA-to-cDNA kit (Applied Biosystems, Life Technologies Corporation, Carlsbad, CA). PCR amplification was done in 96-well plates in 7900 HT real-time PCR system (Applied Biosystems), using TaqMan® Universal PCR Master Mix (Applied Biosystems) and TaqMan® Gene Expression Assays (PRKAA1 Hs01562315_m1, PRKAA2 Hs00178903_m1, Applied Biosystems). All samples were measured in duplicates. Results were normalized to the equal mass of total RNA as well as the Ct values of RPLPO housekeeping control (probe Hs99999902_m1).

### Measurement of intracellular reactive oxygen species

Intracellular reactive oxygen species (ROS) levels were measured using Image-iT LIVE Green Reactive Oxygen Species Detection Kit (Molecular Probes, Life Technologies Europe BV) as previously described [14]. Briefly, the assay is based on 5-(and-6)-carboxy-2′,7′-dichlorodihydrofluorescein diacetate (carboxy-H2DCFDA), a fluorogenic marker that will be cleaved upon the presence of ROS. HCAECs were seeded into 6-well plates. Cells were then incubated for 24 h in EGM medium containing 5% FBS and 2 mM L-glutamine, in the presence or absence of palmitate or vehicle, with or without metformin, AICAR or Compound C. Cells were then washed with Hank’s balanced salt solution (HBSS) before adding 25 μM carboxy-H_2_DCFDA to each well. After 30 minutes of incubation at 37°C, excess probe was removed by washing the cells again with HBSS. HCAECs were then lysed in PBS containing 1% Triton X-100. Carboxy-DCF fluorescence in cell lysates was detected at an excitation/emission wavelength of 495/529 nm using a microplate reader (Tecan Group Ltd., Männerdorf, Switzerland). The fluorescence intensity was normalized against the protein concentration of each individual well.

### Western blot

HCAECs were incubated for up to 24 h in EGM medium containing 5% FBS and 2 mM L-glutamine, in the presence or absence of palmitate or vehicle, with or without metformin, AICAR or Compound C, for the indicated time points. Protein samples from cells were prepared for Western blot analysis using a modified RIPA buffer and Western blot was performed as previously described [15]. Immunoblot analyses were performed using antibodies that recognize phosphorylated AMPK (Cell Signaling Technology, Danvers, MA), total AMPKα1/2 (Abcam, Cambridge, UK), phosphorylated extracellular-signal-regulated kinase 1/2 (ERK1/2) (Millipore Corporatin, Billierica, MA), total ERK 1/2 (Santa Cruz Biotechnology, Heidelberg, Germany), phosphorylated p38 MAPK, total p38 MAPK, phosphorylated c-Jun *N*-terminal kinase (JNK), total JNK, phosphorylated eNOS and total eNOS, cleaved caspase-3 (Cell Signaling Technology, Danvers, MA). Immunoreactive bands were detected using ECL (GE Healthcare, Uppsala, Sweden), imaged with a GelDoc system and quantified with Quantity One software (Bio-Rad Laboratories, Hercules, CA). To verify equal protein loading after imaging, the polyvinylidene difluoride membranes were stained with Coomassie Blue (Bio-Rad Laboratories).

### Statistical analysis

All data are expressed as mean ± SEM. For multiple comparisons, one-way ANOVA or non-parametric Kruskal-Wallis one-way ANOVA on ranks, used as appropriate, the *post-hoc* tests Student–Newman–Keuls and Dunn’s, were used to determine statistical probabilities of differences between groups. A value of p < 0.05 was considered statistically significant.

## Results

### Activation of AMPK protects cells from lipoapoptosis

**Figure 1:**
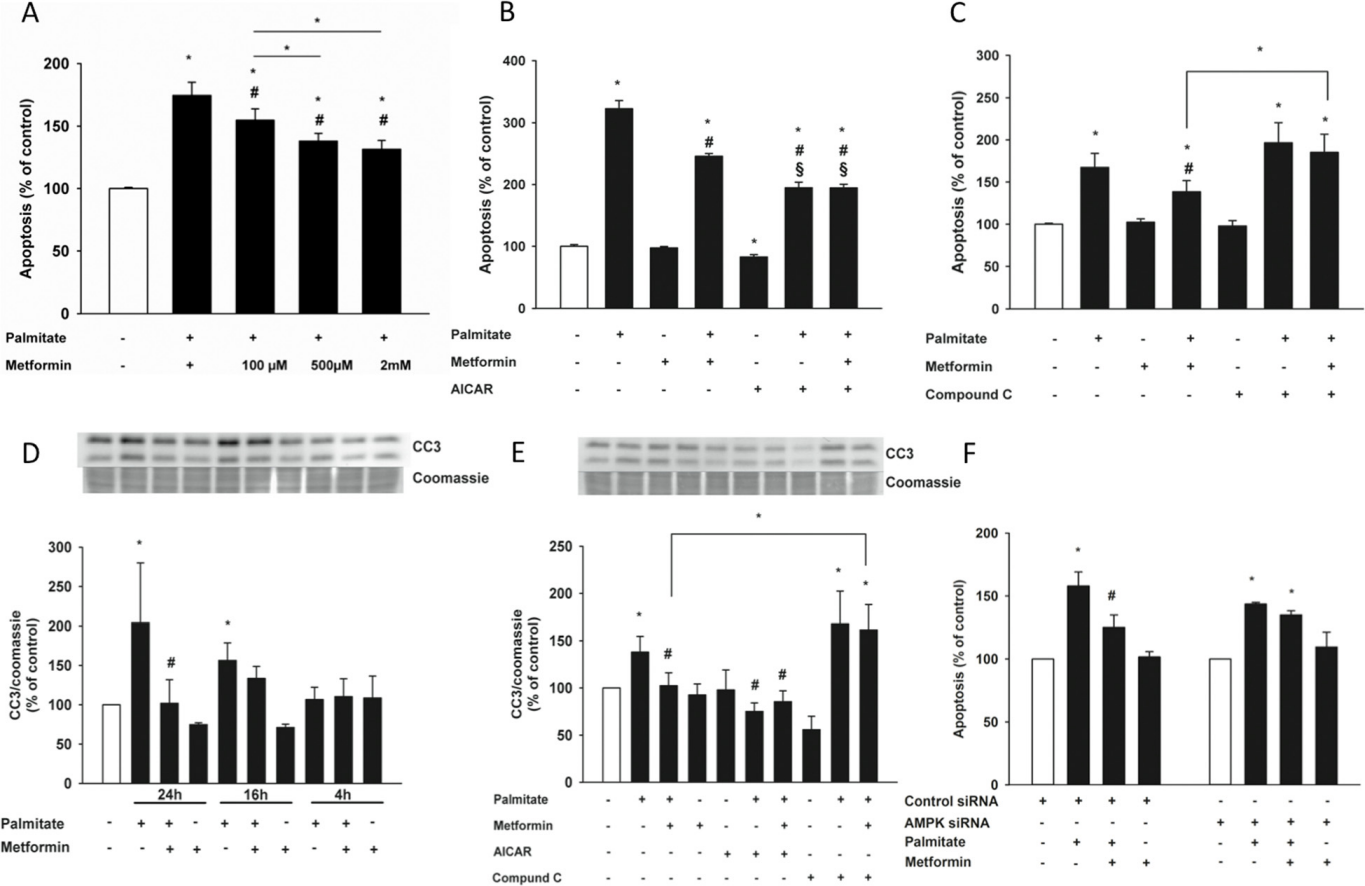
**Activation of AMPK protects human coronary artery endothelial cells from lipoapoptosis.** Cells were incubated in the presence or absence of palmitate (125 μM), metformin (A: 100 μM-2 mM), (B-E: 500 μM), with or without AICAR (200 μM) or Compound C (10 μM). **(A)** Metformin protects cells from lipoapoptosis in a dose-dependent manner after 24 h, an effect that is mimicked by AICAR **(B)** and blocked by Compound C after 24 h of incubation **(C)**. Cleaved caspase 3 (CC3) levels over time **(D)** and in the presence or absence of metformin, AICAR and Compound C after 24 h **(E)**. The protective effect of metformin against palmitate-induced apoptosis is lost upon downregulation of AMPK by siRNA transfection **(F)**. Bars represent mean ± SEM. * p < 0.05 *vs*. control; # p < 0.05 *vs*. palmitate; § p < 0.05 *vs*. metformin + palmitate with Kruskal-Wallis one-way analysis of variance on ranks. n =4-5.

Metformin dose-dependently increased survival of cells exposed to palmitate (Figure 1A). Based on these findings, we used 500 μM of metformin for all subsequent experiments. To further investigate if the protective effect of metformin was AMPK-dependent, we used the specific activator of AMPK, AICAR, and the AMPK inhibitor, Compound C. When cells were co-incubated with AICAR and palmitate, AICAR was able to reproduce the protective effect seen with metformin and actually even further protect the cells against death (Figure 1B). Conversely, co-incubation of cells exposed to palmitate and metformin with Compound C completely abolished the protective effect of metformin (Figure 1C). This was further confirmed with Western blot. Palmitate significantly increased cleaved caspase 3 levels over time, an effect that was significant already after 16 h of treatment (Figure 1D). The increase in cleaved caspase 3 was decreased with metformin treatment, an effect that was mimicked by AICAR but not further reduced when AICAR and metformin were combined. In agreement with these findings, incubation with Compound C completely blocked the effect of metformin on cleaved caspase 3 levels (Figure 1E). The concentrations used herein of Compound C and AICAR were verified to inhibit and activate AMPK, respectively (data not shown). To further support the importance of AMPK, cells were transfected with siRNA against AMPKα1/2. As shown in Figure 1F, the protective effect of metformin was almost completely gone with siRNA treatment against AMPK. However not completely, which might be due to that the AMPK protein levels were down regulated with approximately 40% after 48 h (data not shown).

### Activation of AMPK by metformin and palmitate

**Figure 2:**
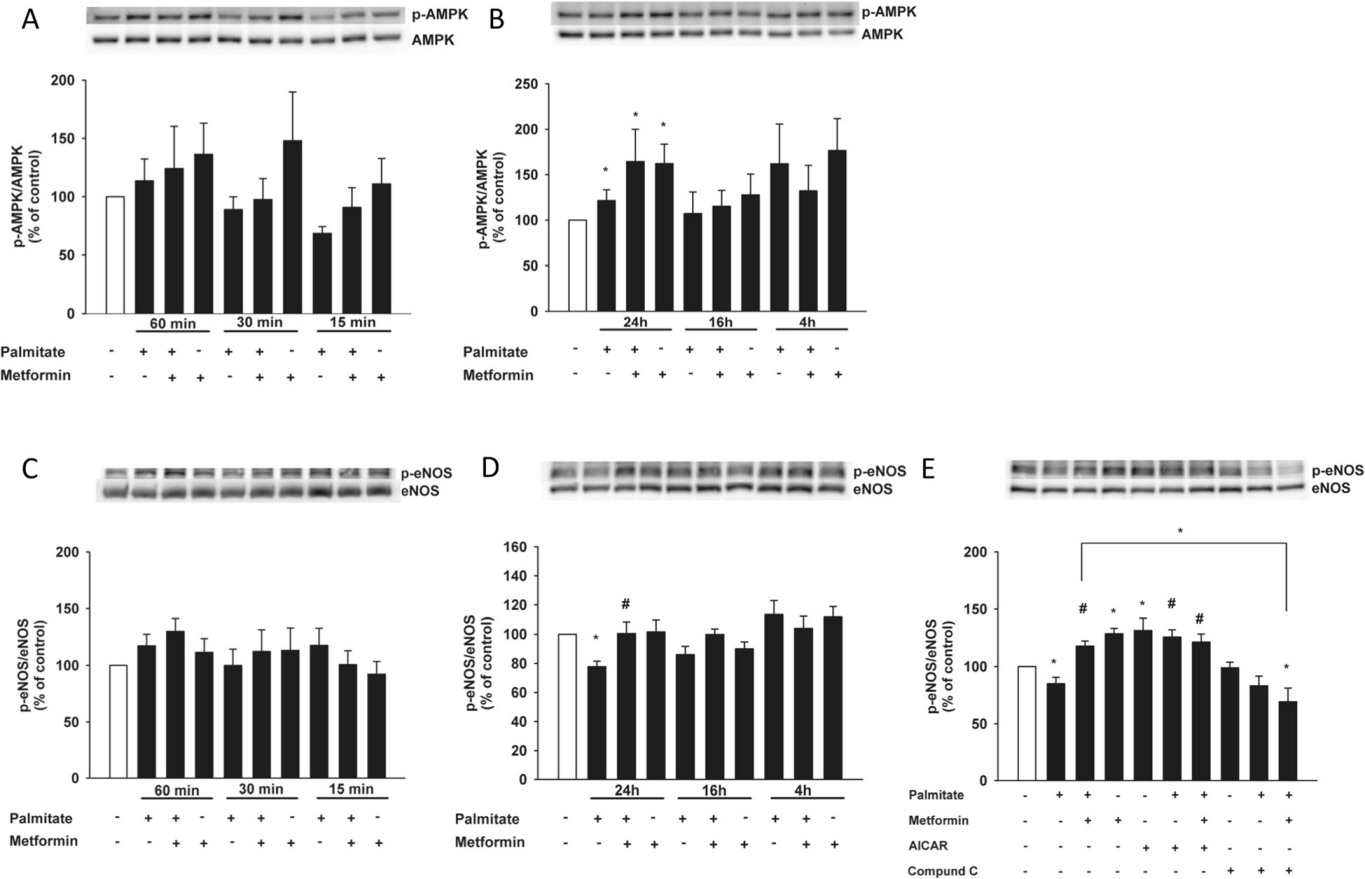
**AMPK is activated by metformin and palmitate over time and AMPK activation restores palmitate-induced eNOS dysfunction.** Cells were incubated in the presence or absence of metformin (500 μM), with or without palmitate (125 μM), for the indicated time points. **(A)** Time course showing phosphorylation levels of AMPK after 60 min, 30 min and 15 min. **(B)** Time course showing phosphorylation levels of AMPK after 24 h, 16 h and 4 h. **(C)** Phosphorylation levels of eNOS after 60 min, 30 min and 15 min. **(D)** Phosphorylation levels of eNOS after 24 h, 16 h and 4 h. **(E)** Phosphorylation levels of eNOS in the presence or absence of metformin (500 μM), AICAR (200 μM) and Compound C (10 μM) after 24 h. Bars represent mean ± SEM. * p < 0.05 *vs*. control with Kruskal-Wallis one-way analysis of variance on ranks **(A, B)** or one-way ANOVA **(C, D, E)**, # p < 0.05 *vs*. palmitate with one-way ANOVA. n =7 **(A, C)**, n = 5 **(B)** n = 6 **(D)**, n = 4 **(E)**.

To further investigate the involvement of AMPK, we studied activation by phosphorylation of AMPK in HCAECs. Cells were incubated with or without metformin and palmitate for the time points indicated in Figure 2. Metformin activated AMPK in a time-dependent manner both in the presence and absence of palmitate; however, the effect was not statistically significant until after 24 h (Figure 2A and B). Palmitate in itself was also able to activate AMPK, albeit to a lesser extent than metformin (Figure 2B).

### Treatment with metformin restores palmitate-impaired phosphorylation of eNOS

Since FFAs impair the bioavailability of NO [7], we studied how exposure to palmitate affected activation of eNOS. Initially palmitate did not affect eNOS phosphorylation at Ser^1177^ (Figure 2C); however, after 24 h of incubation, phosphorylation levels of eNOS were significantly decreased (Figure 2D). Conversely, co-incubation with metformin was able to restore the reduced activation back to control levels (Figure 2D), an effect that was mimicked and even further enhanced by AICAR and completely abolished in the presence of Compound C after 24 h (Figure 2E).

### Involvement of the MAPK pathways

**Figure 3:**
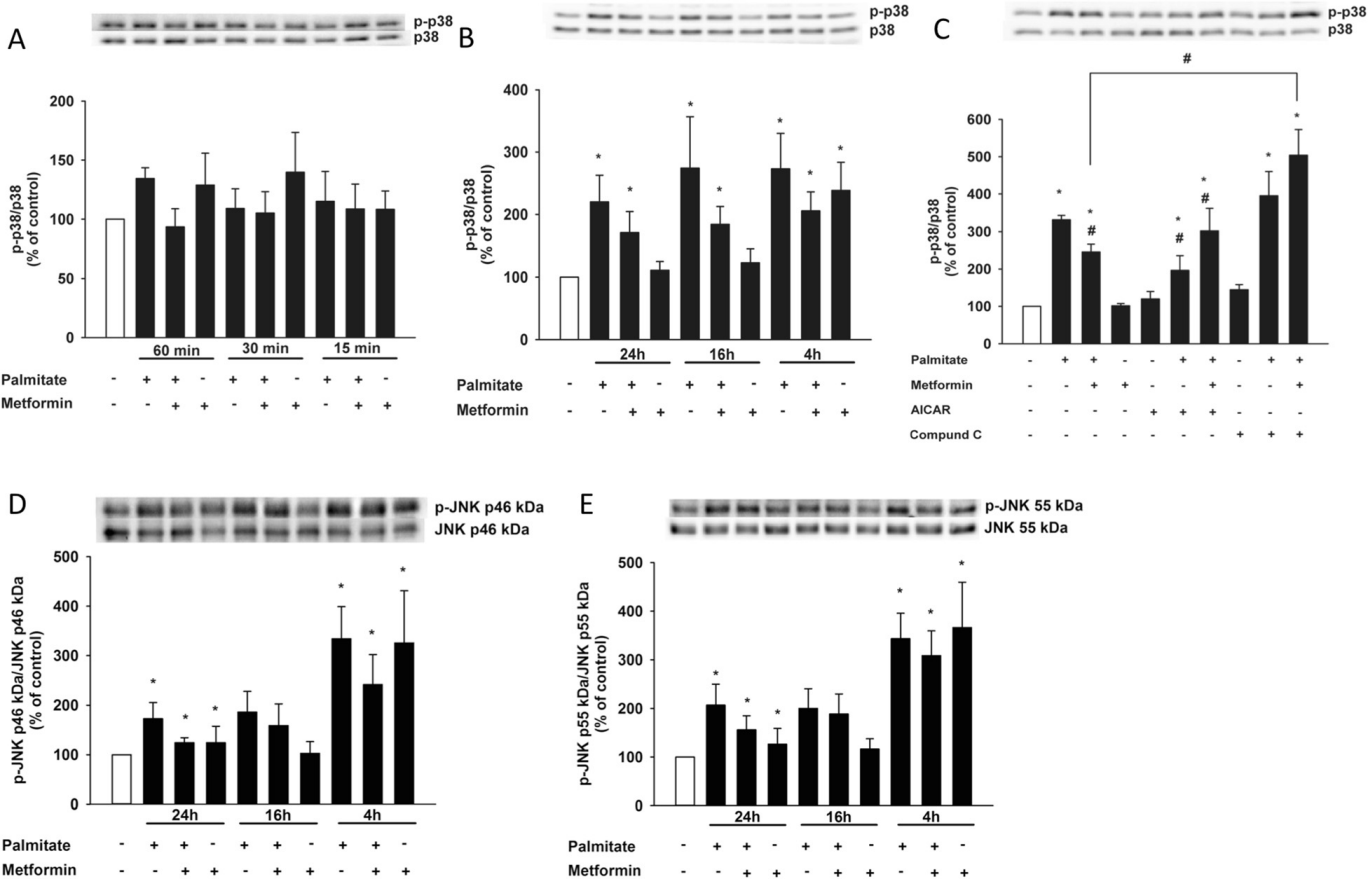
**Palmitate-induced increase of phophorylated p38 MAPK is reduced by metformin treatment.** Cells were incubated in the presence or absence of metformin (500 μM), with or without palmitate (125 μM), for the indicated time points. **(A)** Phosphorylation levels of p38 MAPK after 60 min, 30 min and 15 min. **(B)** Phosphorylation levels of p38 MAPK after 24 h, 16 h and 4 h. **(C)** Phosphorylation levels of p38 MAPK in the presence or absence of metformin (500 μM), AICAR (200 μM) and Compound C (10 μM) after 24 h. **(D and E)** Phosphorylation levels of JNK p46 kDa and p55 kDa after 24 h, 16 h and 4 h. Bars represent mean ± SEM. * p < 0.05 *vs*. control, # p < 0.05 *vs*. palmitate with Kruskal-Wallis one-way analysis of variance on ranks. n =7 **(A)**, n = 5 **(B, D, E)**, n = 4 **(C)**.

In order to elucidate the molecular pathways contributing to metformin’s protective effect, we studied the involvement of the MAPK pathways [8]. HCAECs were incubated with or without metformin and palmitate for the time points specified in Figure 3 We studied how exposure to palmitate affected extracellular-signal regulated kinases (ERK) 1/2, JNK and p38 MAPK over time. Short exposure to palmitate increased phosphorylation levels of p38 MAPK (Figure 3A); however, the effect was not significant until after 4 h of incubation (Figure 3B). Metformin was able to significantly reduce the increase in p38 MAPK activity after 24 h (Figure 3C), an effect that was mimicked by AICAR and blocked by Compound C (Figure 3C). Activation of ERK 1/2 was initially significantly increased by both palmitate and metformin exposure. However, after 16 h both palmitate- and metformin-treated cells displayed a significantly reduced ERK 1/2 activation. There was, however, no significant difference between cells treated with palmitate alone and the combination of and palmitate and metformin for 16 h (data not shown). Phosphorylated JNK levels were significantly increased after 24 h of palmitate exposure; treatment with metformin decreased this activation, albeit the effect did not attain statistical significance (Figure 3D and E).

### The protective effect of metformin involves eNOS and MAPK pathways.

**Figure 4:**
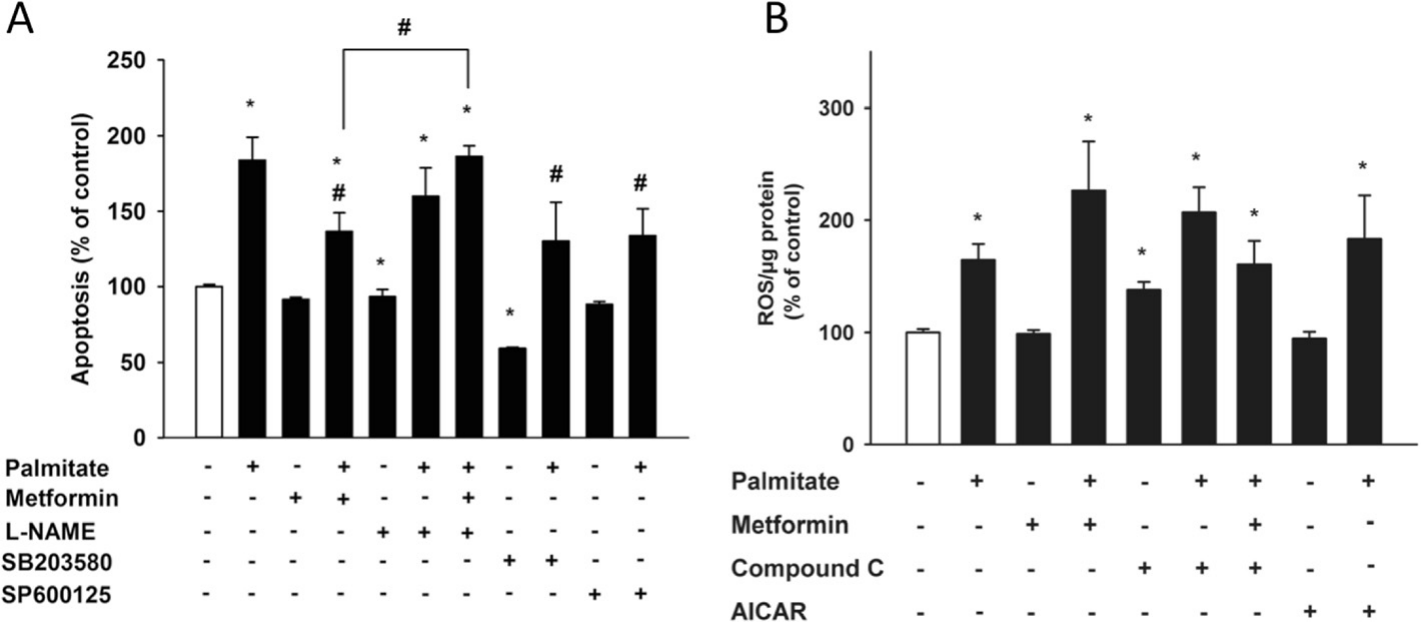
**eNOS and p38 MAPK, but not ROS levels, are involved in the protective effect of metformin.** Cells were incubated for 24 h in the presence or absence of palmitate (125 μM), with or without metformin (500 μM), L-NAME (1 mM), SB203580 (10 μM), SP600125 (5 μM), AICAR (200 μM), Compound C (10 μM) or vehicle. **(A)** Metformin protects cells from lipoapoptosis, an effect that is lost upon co-incubation with L-NAME but is mimicked by SB203580 and SP600125. **(B)** 25 μM of carboxy-H2DCFDA was added to each well for 30 min and ROS levels were subsequently measured. Bars represent mean ± SEM. * p < 0.05 *vs*. control, # p < 0.05 *vs*. palmitate with Kruskal-Wallis one-way analysis of variance on ranks. n = 4 **(A)**, n = 3 **(B)**.

Lastly we set out to further examine the involvement of eNOS in the protective effect of metformin against FFA-induced apoptosis, and the role of p38 MAPK and JNK in lipoapoptosis. We inhibited the eNOS using the arginine analog N_ω_-nitro-L-arginine methyl ester hydrochloride (L-NAME), and addition of L-NAME did not affect the basal levels of palmitate-induced apoptosis; however, upon co-incubation with metformin the protective effect was lost (Figure 4A). Addition of the p38 MAPK inhibitor, SB203580 and JNK inhibitor, SP600125 significantly decreased the lipoapoptosis (Figure 4A), suggesting that p38 MAPK and JNK are important for the apoptosis induced by palmitate and that modulation of these pathways is involved in the protective effect seen with metformin.

### The lipoprotective effect of metformin is not through suppression of ROS

Treatment with AICAR has previously shown to protect bovine aortic endothelial cells from palmitate-induced apoptosis through suppression of ROS [11]. We therefore studied how ROS levels changed in the presence of palmitate, metformin, AICAR and Compound C after 24 h of treatment. As expected, palmitate significantly increased ROS levels; however, neither co-treatment with metformin nor AICAR affected the ROS levels generated by palmitate (Figure 4B).

## Discussion

FFAs are known to induce apoptosis of endothelial cells [8,16] and apoptosis of endothelial cells correlates with an impaired endothelial function [17,18], which is considered a prominent feature of atherosclerosis [19]. Our current results suggest that metformin protects HCAECs from lipoapoptosis caused by long-term exposure of the cells to palmitate, an effect that is AMPK-dependent, involves eNOS and p38 MAPK signaling, but not -- as previously suggested -- suppression of ROS formation.

Although metformin is an old antidiabetic agent and used as first line treatment of T2D, it mechanism of action and other effects are not fully understood. Metformin has recently been shown to have some interesting vasculoprotective effects through improvement of the endothelial glycocalyx barrier in diabetic mice and to inhibit formation of intimal hyperplasia after balloon injury in fructose fed insulin resistant rats [20,21]. Also, we have previously shown that metformin can protect from palmitate-induced apoptosis of HCAECs [22], and we now show that the lipoprotective effect of metformin is conveyed through an AMPK-dependent mechanism. The concentration of metformin in this study is high but it was based on previous publications [10,12,13], in order for us to compare our results to others’ findings. In line with our data, activation of AMPK by AICAR has previously been demonstrated to protect bovine aortic endothelial cells from lipoapoptosis [11] and human umbilical vein endothelial cells from high glucose-induced apoptosis [13]. As expected, metformin caused a robust phosphorylation of AMPK, which was sustained in the presence of palmitate. We also detected a slight increase in AMPK phosphorylation upon incubation with palmitate, a phenomenon previously reported in myocytes [23]. A possible explanation for this finding is that palmitate is known to cause oxidative stress [11] and oxidative stress, *i.e.* peroxynitrite, has in turn been shown to activate AMPK without strict AMP dependency [24,25]. Also, activation of AMPK leads to an increased lipid oxidation in cells [26], and palmitate in combination with AMPK activation enhances this oxidation even further [27]. We also noticed, however the effect was not statistically significant, that treatment with only metformin in general gave a better response of the cells to metformin. This might be due to the condition of the cells, since palmitate treatment induces apoptosis probably making the cells respond less efficiently. Also, we have not investigated how palmitate affects the phosphorylation of AMPK at Ser-485/497, which was recently discovered to reduce phosphorylation of AMPK at Thr-172 and thus serves as a negative regulator of AMPK activity [28].

Elevated plasma levels of FFAs are often seen in T2D patients [8], and FFAs have been shown to impair eNOS activity [29] and to induce endothelial dysfunction in humans [30]. Metformin, through AMPK activation, is able to phosphorylate eNOS and thus increase NO production [10] and we have previously described the importance of eNOS in protection against lipotoxicity of HCAECs [16]. We therefore set out to study the effects of palmitate on eNOS activity. Long-term exposure of endothelial cells to palmitate decreased eNOS phosphorylation [29], an effect that was countered by metformin. This effect was completely abolished when metformin was co-incubated with Compound C, while AICAR -- on the other hand -- mimicked metformin. Adding support to the importance of eNOS, co-incubation of metformin and the eNOS inhibitor L-NAME almost completely blocked the protective effect of metformin. Taken together, this suggests that metformin protects HCAECs from lipoapoptosis through an AMPK-dependent activation of eNOS.

JNK and p38 MAPK are stress-inducible kinases that are known to be involved in apoptosis signaling in a variety of cells [16,31,32] and p38 MAPK in particular has been shown to be important for palmitate-induced apoptosis in HCAECs [8]. Metformin was able to decrease the induced phosphorylation of p38 MAPK, an effect that was AMPK-dependent; the effect of metformin was also mimicked by the p38 MAPK inhibitor SB203580. Activation of JNK was increased by FFA exposure, and even though metformin was able to decrease this phosphorylation after 24 h, the effect was not statistically significant. Incubation with a JNK inhibitor decreased the lipoapoptosis, thus confirming the importance of JNK activation for palmitate-induced apoptosis of HCAECs. Taken together, our data suggest that the partial protective effect imposed by metformin is mediated through modulation of p38 MAPK activation, downstream of AMPK signaling.

JNK and p38 MAPK are -- among other stressors -- activated by ROS [33] and since we did detect an increase in ROS, we hypothesized that metformin might reduce ROS as part of its lipoprotective effect. Surprisingly, we could not detect a difference in ROS levels, neither with metformin nor AICAR treatment. Contrary to what we found in our study, Kim and colleagues suggested that the lipoprotective effect of AICAR is mediated by suppression of ROS [11] and others have also shown that metformin can reduce high glucose-induced ROS [34]. The reason for this discrepancy is hard to pin down. But in speculation, palmitate increases lipid oxidation and can also lead to ceramide formation, two processes that generate ROS [35]. Additionally, simultaneous activation of AMPK increases fatty acid oxidation even further [27]. The unchanged levels of ROS seen with metformin might therefore reflect an increased lipid oxidation, which contributes to its protective effect. Very recently, it was shown that treatment with metformin, in a model of the organism Caenorhabditis elegans, increased ROS and β-oxidation. This effect was coupled to an increased longevity of the worm, in which metformin-induced production of reactive species increases the overall life expectancy [36]. Also, small fluctuations of ROS have interestingly been suggested to play an obligatory role in intracellular signaling [37].

## Conclusion

To the best of our knowledge, this is the first report showing that metformin can protect HCAECs from lipoapoptosis, through an AMPK-dependent mechanism, which recover eNOS activity and decrease p38 MAPK signaling. Our data suggest that metformin might directly improve endothelial dysfunction observed under diabetic conditions irrespective of its glycemic effects. This finding is potentially clinically relevant, since metformin is used as first line treatment for patients with T2D, a patient group that is rapidly increasing worldwide and carries a high burden of cardiovascular disease.

## Abbreviations

AICAR: 5-aminoimidazole-4-carboxamide-1-β-D-ribofuranoside
AMPK: AMP-activated protein kinase
BSA: Bovine serum albumin
ELISA: Enzyme-linked immunosorbent assay
eNOS: Endothelial nitric oxide synthase
ERK: Extracellular signal-regulated kinase
FBS: Fetal bovine serum
FFA: Free fatty acid
HCAEC: Human coronary artery endothelial cell
JNK: c-Jun N-terminal kinase
L-NAME: N_ω_-nitro-L-arginine methyl ester hydrochloride
NO: Nitric oxide
ROS: Reactive oxygen species
T2D: Type 2-diabetes
UKPDS: The UK Prospective Diabetes Study
